# Complete Genome Sequences of Two Apple Stem Grooving Viruses in Cnidium officinale in Korea

**DOI:** 10.1128/mra.00902-22

**Published:** 2022-12-07

**Authors:** Da-Som Lee, Hong-Kyu Lee, So-Yeon Kim, Bo-Ram Kwon, Hee-Ji Yang, Chung Youl Park, Su-Heon Lee

**Affiliations:** a Department of Applied Biosciences, Kyungpook National University, Daegu, Republic of Korea; b Division of Wild Plant Seed Research, Baekdudaegan National Arboretum, Bonghwa, Republic of Korea; Katholieke Universiteit Leuven

## Abstract

We determined the whole-genome sequences of two apple stem grooving viruses (ASGV) detected in infected *Cnidium officinale* plants. The analyzed ASGV genomes were 6,494 nucleotides long and encoded two overlapping open reading frames. Phylogenetic analysis revealed the two ASGV isolates to be most closely related to the ASGV isolate Xinjiang-3.

## ANNOUNCEMENT

*Apple stem grooving virus* (ASGV) is a member of the genus *Capillovirus* and belongs to the family *Betaflexiviridae* (1). The virus has a polyadenylated positive-sense single-stranded RNA genome consisting of two overlapping open reading frames (ORFs) that encode a polyprotein and a movement protein (MP) (2).

To date, six viruses have been identified in Cnidium officinale (3–7). However, the first published South Korean ASGV isolate recovered from *C. officinale* has only been partially sequenced (GenBank accession numbers MW889883 and MW889884). Thus, the present study aimed to decipher the whole-genome sequences of two ASGV isolates recovered from *C. officinale*.

In this study, 39 *C. officinale* leaf samples and 3 *Ligusticum chuanxiong* leaf samples were obtained in Yeongju, South Korea, in 2018. Total RNA was extracted from the pool using an easy-spin total RNA extraction kit (iNtRON, Daejeon, South Korea). rRNA was eliminated using the Ribo-Zero rRNA removal kit (Illumina, San Diego, CA, USA), and an RNA library was generated using the TruSeq stranded total RNA low-throughput (LT) sample prep kit (Illumina) (8). High-throughput RNA sequencing (RNA-seq) was conducted by Macrogen Inc. (Seoul, South Korea) on an Illumina NovaSeq 6000 platform (Illumina) using paired-end 2 × 101-bp reads. The raw reads were analyzed using FastQC v0.11.7, and low-quality reads, adaptor sequences, and contaminant DNA were trimmed using Trimmomatic v0.38, followed by *de novo* assembly using the Trinity vr20140717 program. The assembled contigs were analyzed using DIAMOND software (NCBI blastn and blastx) with a default E value cutoff of 1.0E–5. All tools were run with default parameters unless otherwise specified.

The *de novo* transcriptome assembly of the 100,116,894 raw reads resulted in 75,798 contigs. Of these, 10 ASGV contigs with lengths ranging from 305 to 3,202 nucleotides (nt) and sequence identities of 78.99% to 96.12% (query coverage, 82% to 100%) to the ASGV isolate found under GenBank accession number NC_001749 were obtained using BLAST v2.4.0+ (https://blast.ncbi.nlm.nih.gov/Blast.cgi) (9).

Eight primer sets were designed based on the contig sequences to determine the whole-genome sequence of ASGV using two randomly selected ASGV-positive samples (Table 1). The terminal sequences of both genomes were determined with specific primer sets using the 5′/3′ rapid amplification of cDNA ends (RACE) system (Invitrogen, Carlsbad, CA, USA). Accordingly, 24 positive amplicons were cloned into the RBC T&A cloning vector (RBC Bioscience, Taiwan) and sequenced using Sanger sequencing (BioFact, Daejeon, South Korea).

**TABLE 1:** Primers used in PCR amplification to obtain the whole-genome sequences of apple stem grooving virus isolates BH and YY

Set no.	Primer name	Oligonucleotide sequence (5′ to 3′)	Amplicon size (bp)
1	ASGV-F1	ACC ACA GAC GAG ATT GAA AAG	1,180
	ASGV-R1	CTT CAA TTC CTT TGG TGG CC	
2	ASGV-F1-Cn	CAC CCC AGA AGA TCA AGA AC	1,209
	ASGV-R1-Cn	CCA ATA GCC TCC GCT ATA TC	
3	ASGV-F2	GGC CAC CAA AGG AAT TGA AG	1,568
	ASGV-R2	GAC CAA ACC TGT ACC CTT GG	
4	ASGV-F3	GGA GAT CCA CTT CAG CTA AG	1,373
	ASGV-R3	GTA ACG AAG CTG TCC AGA CA	
5	ASGV-F3-Cn	GTT GGA CAA AGC GCA TGA CA	1,352
	ASGV-R3-Cn	AGC CGT CAA ATG CGC TTG CA	
6	ASGV-F4	GAG GCA AAA GCT GGT CAA AC	931
	ASGV-R4	GTT GAC TCG ACC TCC TTG AG	
7	ASGV-F5	CTA CAG ATT AGG TGA GAG GC	980
	ASGV-R5	GAG AGG GAC CTA AAT CCT CT	
8	ASGV-F6	GGA GTT GCG TAC AGA TGC G	1,009
	ASGV-R6	ACT CTC CGA ACC TGC CTC G	
5′ RACE	5RACE-R1	GGC AAC TGA CTT GAA GGA AG	NA
	5RACE-R	AGG ACT GAG TGC ATC TTG AG	NA
3′ RACE	3RACE-F1	GAA CCG TCT AAC ACC TGA TG	NA
	3RACE-F2	CAG GTG ATT GAC AGG ATG AC	NA

**FIG 1:**
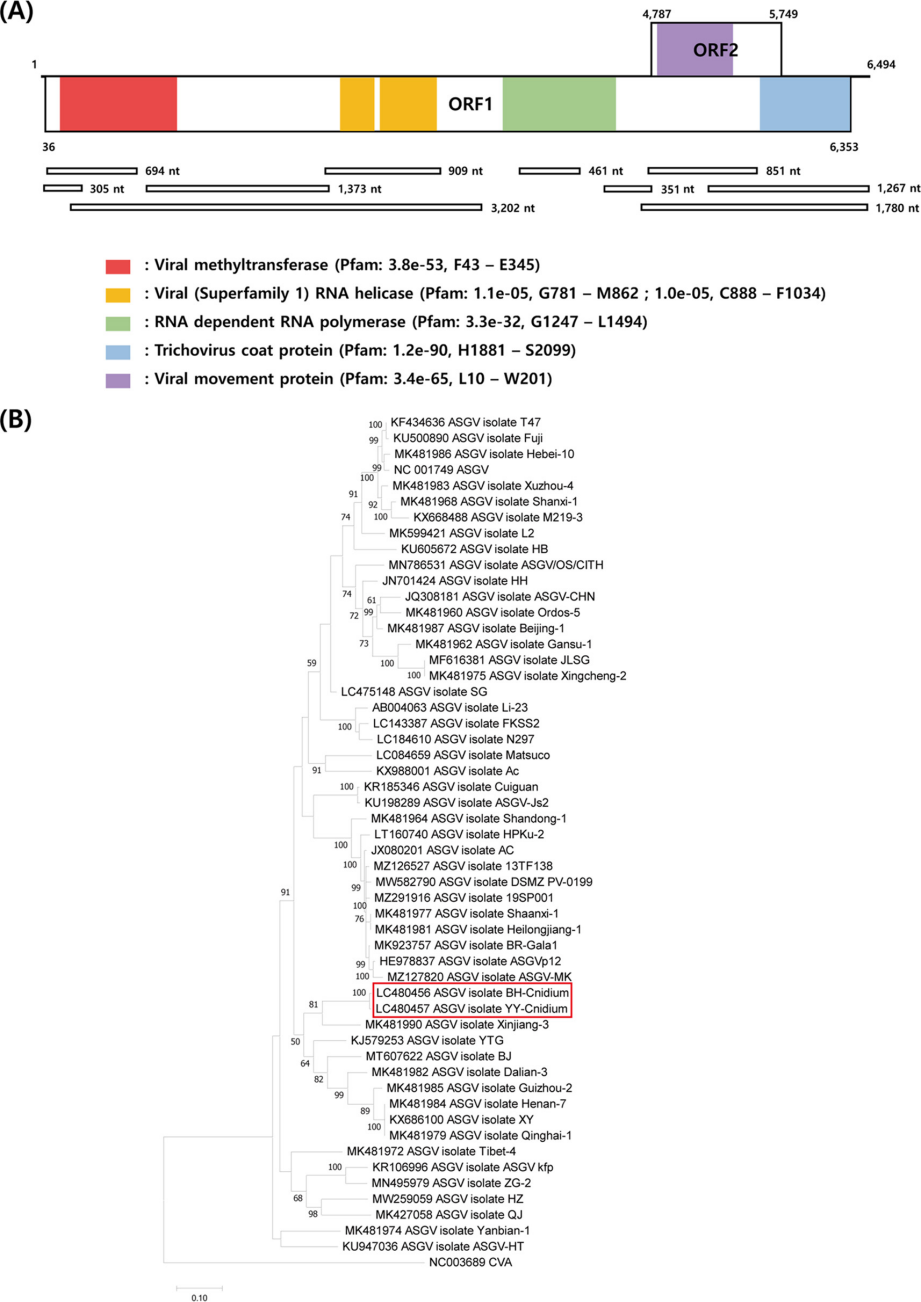
Genome organization and phylogenetic analysis of the apple stem grooving virus (ASGV) isolates BH and YY. (A) Schematic representation of the ASGV isolates BH and YY. The ten white boxes below the genome organization schematic indicate the contigs produced by high-throughput RNA sequencing. ASGV isolates BH and YY contain two overlapping open reading frames. Conserved domains/motifs were identified using Pfam with effective values. (B) Phylogenetic tree constructed using the complete genome sequences of the two ASGV isolates (BH and YY) from the present study and 51 other ASGV isolates using the maximum-likelihood method based on the Jones-Taylor-Thornton model in MEGA v11 software. A total of 1,000 bootstrap replications were performed.

The whole-genome sequences of the two ASGV isolates—BH and YY—were 6,494 nt long, with GC contents of 42.73% and 42.51%, respectively, and comprised two overlapping ORFs, similar to those of the canonical ASGV isolate genomes (NCBI protein accession numbers P36309 and P36698). ORF1 and ORF2 encoded a polyprotein (36 to 6,353 nt) and MP (4,787 to 5,749 nt), respectively. Conserved domains and motifs, including viral methyltransferase, viral RNA helicase, RNA-dependent RNA polymerase, coat protein, and MP, were identified using the online Pfam search (http://pfam.xfam.org/) (Fig. 1A). Phylogenetic analysis was performed using the whole-genome sequences of the two ASGV isolates from the present study and other reported ASGV isolates using MEGA v11 software. The results showed that the whole-genome sequences of the ASGV isolates BH and YY were most closely related to that of ASGV Xinjiang-3, isolated from apple trees (GenBank accession number MK481990), with 85.24% nucleotide identity (Fig. 1B).

### Data availability.

The whole-genome sequence of ASGV isolates BH and YY have been deposited at GenBank under accession numbers LC480456 and LC480457, respectively. The raw data have been deposited in the Sequence Read Archive under BioProject accession number PRJNA868815.

## References

[1] Shim H-K, Hwang K-H, Shim C-K, Son S-W, Kim D-G, Choi Y-M, Chung Y-J, Kim D-H, Jee H-J, Lee S-C. 2006. Molecular characterization of apple stem grooving virus isolated from *Talaromyces flavus*. Plant Pathol J 22:260–264. doi:10.5423/PPJ.2006.22.3.260.

[2] Yoshikawa N, Sasaki E, Kato M, Takahashi T. 1992. The nucleotide sequence of apple stem grooving Capillovirus genome. Virology 191:98–105. doi:10.1016/0042-6822(92)90170-T. 1413530

[3] Belete MT, Igori D, Kim SE, Lee S-H, Moon JS. 2022. Complete genome sequence of Cnidium virus 1, a novel betanucleorhabdovirus infecting *Cnidium officinale*. Arch Virol 167:973–977. doi:10.1007/s00705-021-05348-9. 35112199

[4] Chung BN, Kwon S-J, Yoon J-Y, Cho I-S. 2022. First report of *Cnidium officinale* as a natural host plant of apple stem grooving virus in South Korea. Plant Dis 106:338. doi:10.1094/PDIS-04-21-0781-PDN. 34319765

[5] Honma H, Tsushima D, Kawakami H, Fujihara N, Tsusaka T, Kawashimo M, Nishimura T, Fuji S. 2019. Complete nucleotide sequence of a new Potexvirus, “Cnidium virus X,” isolated from *Cnidium officinale* in Japan. Arch Virol 164:1931–1935. doi:10.1007/s00705-019-04261-6. 31011816

[6] Igori D, Lee H-K, Yang H-J, Lee D-S, Kim S-Y, Kwon B, Oh J, Kim T-D, An C, Moon J-S, Lee S-H. 2020. First report of the cycas necrotic stunt virus infecting *Cnidium officinale* in South Korea. Plant Dis 104:3275–3275. doi:10.1094/PDIS-01-20-0092-PDN.

[7] Yoo RH, Zhao F, Lim S, Igori D, Kim S-M, An T-J, Lee S-H, Moon JS. 2015. The complete genome sequences of two isolates of Cnidium vein yellowing virus, a tentative new member of the family Secoviridae. Arch Virol 160:2911–2914. doi:10.1007/s00705-015-2557-1. 26282235

[8] Lee DH, Kim J, Han JS, Lee J-H, Lee B, Park CY. 2020. Detection, isolation and characterization of cucumber mosaic virus from *Pseudostellaria heterophylla* in Korea. J Plant Biotechnol 47:150–156. doi:10.5010/JPB.2020.47.2.150.

[9] Cameron M, Williams HE, Cannane A. 2004. Improved gapped alignment in BLAST. IEEE/ACM Trans Comput Biol Bioinform 1:116–129. doi:10.1109/TCBB.2004.32. 17048387

